# Shifting patterns and competing explanations for infectious disease priority in global health agenda setting arenas

**DOI:** 10.1093/heapol/czae035

**Published:** 2024-05-16

**Authors:** Stephanie L Smith, Rakesh Parashar, Sharmishtha Nanda, Jeremy Shiffman, Zubin Cyrus Shroff, Yusra Ribhi Shawar, Dereck L Hamunakwadi

**Affiliations:** School of Public and International Affairs, Virginia Tech, 900 N. Glebe Rd, Arlington, VA 22203, USA; Global Business School for Health, University College London, UCL East Marshgate, 7 Sidings Street, London E20 2EA, United Kingdom; Alliance for Health Policy and Systems Research, World Health Organization, # C 1021, Sushant Lok 1, Gurgaon, Delhi, India; Independent Consultant, C 1021, Sushant Lok-1, Gurgaon, NCR 122002, India; International Health, Johns Hopkins University, 615 N. Wolfe St. E8539, Baltimore, MD 21205, USA; School of Advanced International Studies, Johns Hopkins University, 615 N. Wolfe St. E8539, Baltimore, MD 21205, USA; Alliance for Health Policy and Systems Research, World Health Organization, Avenue Appia 20, Geneva 1211, Switzerland; International Health, Johns Hopkins University, 615 N. Wolfe St. E8539, Baltimore, MD 21205, USA; School of Public and International Affairs, Virginia Tech, 900 N. Glebe Rd, Arlington, VA 22203, USA

**Keywords:** Global health, priority, agenda setting, arenas model, health policy, infectious disease, communicable disease, neglected disease

## Abstract

The highly decentralized nature of global health governance presents significant challenges to conceptualizing and systematically measuring the agenda status of diseases, injuries, risks and other conditions contributing to the collective disease burden. An arenas model for global health agenda setting was recently proposed to help address these challenges. Further developing the model, this study aims to advance more robust inquiry into how and why priority levels may vary among the array of stakeholder arenas in which global health agenda setting occurs. We analyse order and the magnitude of changes in priority for eight infectious diseases in four arenas (international aid, scientific research, pharmaceutical industry and news media) over a period of more than two decades in relation to five propositions from scholarship. The diseases vary on burden and prominence in United Nations Sustainable Development Goal 3 for health and well-being, including four with specific indicators for monitoring and evaluation (HIV/AIDS, tuberculosis, malaria, hepatitis) and four without (dengue, diarrhoeal diseases, measles, meningitis). The order of priority did not consistently align with the disease burden or international development goals in any arena. Additionally, using new methods to measure the scale of annual change in resource allocations that are indicative of priority reveals volatility at the disease level in all arenas amidst broader patterns of stability. Insights around long-term patterns of priority within and among arenas are integral to strengthening analyses that aim to identify pivotal causal mechanisms, to clarify how arenas interact, and to measure the effects they produce.

Key messages

- Priority in arenas does not dependably align with international development goals.
- Priority does not consistently align with the global disease burden in any arena.
- New methods for measuring the scale of change in priority levels are introduced.
- Patterns of disease priority are highly unstable in all arenas.

## Background

Gaining status on the global health agenda holds promise for widespread policy adoption and resource allocations that result in improved and more equitable public health outcomes (Koplan *et al*., 2009; Moran *et al*., 2012). Smith *et al*. (2021) introduced an arenas model to address challenges to conceptualizing and measuring the global health agenda in the highly decentralized governance context. According to the pluralistic arenas model, problems are defined and compete for resource allocations in a wide range of overlapping and interacting stakeholder arenas that are central to their address, including but not limited to governments, private industries (e.g. pharmaceutical, unhealthy food and beverages), international aid, media, scientific research and civil society (Hilgartner and Bosk, 1988; Shiffman *et al*., 2010; Smith and Gorantla, 2021; Smith *et al*., 2021; Smith, 2023). An arenas model focuses inquiry on who among key collectivities of actors prioritizes which issues, how, when and why, and the intersecting webs of socio-political dynamics that shape agendas. An early application of the model, shows that coronaviruses overtook established (HIV/AIDS), emergent (diabetes) and rising (Alzheimer’s disease) global health issues in multiple arenas in 2020, reflecting impacts of the COVID-19 pandemic as a focusing event (Smith *et al*., 2021). This study takes steps to advance more robust inquiry into how and why priority levels may vary within and among global health agenda setting arenas.

This study is part of the Global Health Agendas Project, a research programme formed to advance concepts and measurement of the global health agenda (Shiffman *et al*., 2010; Smith and Gorantla, 2021; Smith *et al*., 2021; Smith, 2023; Parashar *et al*., working paper). This study extends this emergent body of work developing an arenas model for global health agenda setting by analysing infectious disease priority levels in a subset of arenas (international aid, scientific research, pharmaceutical industry and news media) in relationship to propositions informed by policy analysis scholarship. It also explores long-term patterns of stability and large-scale change. Novel research methods used in the study position scholars and other analysts to conduct more robust analysis of causal dynamics and priorities in health agenda setting arenas.

The exploratory study is guided by two research questions: (1) Does the order of disease priority in arenas align with relative mortality burdens or status in major international development goals—prominent rational and socially constructed explanations for differential priority? And (2) Do patterns of annual change in priority within and among arenas reflect relatively small in scale change (stability) or are they more volatile in nature? Alternatively, do these patterns reflect findings of many scholars showing that policy change is typically small-scale and occasionally interrupted by short periods of large-scale change (e.g. punctuated equilibria; Baumgartner, Jones and Mortensen, 2018; Baumgartner and Jones, 1993)? We examine five propositions from scholarship on how disease priority levels may be determined (Propositions 1–3) and the patterns they might exhibit (Propositions 4–5). The study covers eight infectious diseases selected for variance on mortality burden and prominence among major international development goals in four global health agenda setting arenas for which longitudinal data are available.

Proposition 1: Disease priority levels are likely to align with global disease burden.

**Figure 1.**
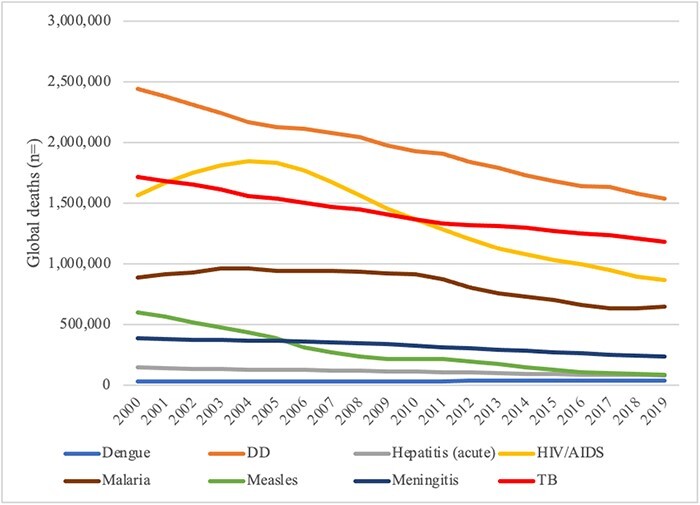
Global mortality burden, 2000–2021

Source: Global Burden of Disease Collaborative Network (2020).

Informing our first proposition from scholarship, a rational model prescribes and predicts alignment between disease burden and the order of global health priorities (Shiffman *et al*., 2002). The diseases selected for this investigation vary substantially in terms of global mortality burden (Figure 1). The overall toll of diarrhoeal diseases (DD)—one of the leading causes of death among all age groups globally—surpassed tuberculosis (TB) and HIV/AIDS each by a 1.4 to 1 ratio, malaria by 2.4 to 1, meningitis by 6.1 to 1, measles by 7.2 to 1, hepatitis by 17.7 to 1 and dengue by 63.6 to 1 ratio between 2000 and 2019 (Global Burden of Disease Collaborative Network, 2020). A rational order of priority would descend from the highest to lowest burden disease.

Proposition 2: Disease priority levels are likely to align with major international development goals.

Major international development goals establish norms—socially constructed expectations for the behaviour of concerned actors to back a cohesive set of priorities (Finnemore and Sikkink, 1998; Shiffman, 2009). Particularly powerful ‘supernorms’ like the Millennium Development Goals (MDGs) and Sustainable Development Goals (SDGs) are especially suggestive of alignment on the basis of norm strength (Fukuda-Parr and Hulme, 2011). Prominence under United Nations MDG 6 (to combat HIV/AIDS, malaria and other diseases) and SDG 3 for health and well-being suggests an order of priority that differs from one based on mortality burden (Table 1). HIV/AIDS and malaria headlined MDG 6 and had dedicated targets, a strong position among the international development goals. HIV/AIDS (Indicator 3.3.1), TB (Indicator 3.3.2), malaria (Indicator 3.3.3) and hepatitis (Indicator 3.3.4) are prominent under SDG 3, Target 3 infectious diseases, with dedicated monitoring and evaluation indicators. Dengue and DD, respectively, are unnamed but well-recognized among neglected tropical diseases (NTDs, SDG Indicator 3.3.5) and waterborne diseases (SDG 3.3 but no indicator) (United Nations Statistics, n.d.a). Measles and meningitis are less prominent, relegated to ‘other’ categories under SDG 3.3.

**Table 1.** Diseases by international development goal status and norm strength

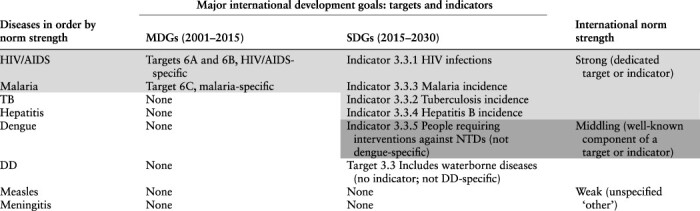

Note: Targets and indicators are linked to monitoring and accountability mechanisms.

Sources: United Nations Statistics, n.d.b; n.d.a.

Proposition 3: Disease priorities are likely to vary by stakeholder arena.

A third and contradictory proposition rejects universal application of rational and constructivst rationales on global priority ordering; instead, this proposition advances that disease priorities are likely to vary by stakeholder arena. Under this assumption, the agenda is unlikely to be so neatly ordered or uniform in a highly decentralized global health governance setting in which no single institution or group has overarching authority (Fidler, 2007; Youde, 2018). Within and among the various stakeholder arenas that are central to global health governance, an array of more and less powerful actors, rational interests (financial and security), ideas (beliefs, values, causal stories and other issue frames), institutional (rules, policies, norms) and other factors interact to shape differing sets of priorities (Baumgartner and Jones, 1993; Shiffman *et al*., 2002; Shiffman and Smith, 2007; Bump *et al*., 2013; Walt and Gilson, 2014; Shearer *et al*., 2016; Smith and Shiffman, 2018). And, proponents shop for receptive venues (Baumgartner and Jones, 1993), increasing the likelihood of earlier issue adoption in some arenas than others. Convergence among a number of factors across multiple arenas may nonetheless sometimes open windows of opportunity to affect large-scale change (Kingdon, 1995; Shiffman and Smith, 2007; Fox *et al*., 2015). Such dynamics are not investigated but are likely reflected in the patterns revealed by this study.

Proposition 4: ‘Overall’ patterns of resource allocations from arenas are likely to be characterized by mostly small-scale change that is occasionally punctuated by large-scale shifts.

 

Proposition 5: Patterns of resource allocations from arenas ‘to specific diseases’ are likely to be characterized by mostly small-scale change that is occasionally punctuated by large-scale shifts.

Turning to patterns of priority over time and towards our fourth and fifth propositions, priority or status on the global health agenda is indicated by levels of various kinds of stated, institutional, financial and other resource commitments allocated to address issues (Shiffman and Smith, 2007; Fox *et al*., 2011; 2015; Bump *et al*., 2013; Walt and Gilson, 2014). In an arenas model, relative priority is indicated by the meaningful allocation of specific kinds of resources that are offered by and demanded of relevant collectivities of actors comprising agenda setting arenas (Hilgartner and Bosk, 1988; Shiffman *et al*., 2010; Smith and Gorantla, 2021; Smith *et al*., 2021; Smith, 2023; Parashar *et al*., working paper). For instance, priority is indicated by levels of development assistance for health (DAH) in the international aid arena, product development and offerings in industry arenas, bibliographic trends in the scientific research arena, and headlines in the news media arena (see also Parashar et al.’s [working paper] discussion of alternative priority indicators and measurement). Analysis of priorities and causal dynamics within and among global health agenda setting arenas remains in an early phase of development.

A sizeable volume of mainstream agenda setting scholarship shows that government agendas are largely stable and characterized by series of incremental changes over long periods of time that are occasionally ‘punctuated’ by sizeable shifts (Baumgartner, Jones and Mortensen, 2018; Baumgartner and Jones, 1993; Dowding *et al*., 2016). Punctuations are defined by deviation from the status quo or typical patterns (Jones *et al*., 1998; Jordan, 2003; Breunig and Koski, 2006). Patterns of punctuated equilibrium have also been observed in research on global health security regimes (Hoffman, 2010), international aid for health (Martin and Streams, 2015) and priority for infectious diseases (Shiffman *et al*., 2002).

We explore the nature of long-term patterns of change in priority within and among several arenas for global health agenda setting. We explore whether, over an extended period of time, these patterns are consistent with patterns of punctuated equilibria or characterized by persistently small-scale or more volatile patterns of change at the level of an arena’s ‘overall allocations of a specific kind of resource’ (Proposition 4) and as that resource is directed ‘to specific diseases’ (Proposition 5). Given the large body of research finding patterns of primarily small-scale incremental change that is occasionally punctuated by major change, we expect the same at the overall and disease levels for most arenas. However, persistent volatility may be typical in some arenas (e.g. news media driven by daily news cycles), which would require different parameters for identifying unusually large-scale change. Studying long-term patterns of priority and change within and among stakeholder arenas is needed to strengthen causal analyses—doing so can help to identify those pivotal levers that precipitate divergence from the status quo (Baumgartner and Jones, 1993; Dowding *et al*., 2016).

## Methodology

This study draws upon other analyses measuring the status of problems in global health agenda setting arenas but is distinguished from them by its novel inquiry into competing explanations for differences in priority levels and long-term patterns of (in)stability (Shiffman *et al*., 2010; Smith and Gorantla, 2021; Smith *et al*., 2021; Smith, 2023; Parashar *et al*., working paper). To build a foundation for our theoretically informed analysis, the first step is to ‘measure priority levels’ in key global health agenda setting arenas—our data and methods largely follow Smith *et al.’s* (2021) original analysis in this. However, we expand from comparison of change in priority levels in the context of the COVID-19 pandemic shock (2019–2020) to exploring more typical ‘patterns of change and scale’ in priority year-to-year by arena over 22 years (2000 through 2022) (see also Parashar *et al*., working paper). The indicators (DAH, clinical trials, research and news media publishing) used in this analysis are rooted in previous scholarship analysing priority for global health issues (Bump *et al*., 2013; Footman *et al*., 2014; Hudacek *et al*., 2011; Pitt *et al*., 2018; Ravishankar *et al*., 2009; Smith *et al*., 2021; Smith and Gorantla, 2021).

Data collection and analysis procedures were tailored to each arena. In the ‘international aid arena’, DAH is a major indicator of priority (Ravishankar *et al*., 2009; Pitt *et al*., 2018). Allocations of such budgetary commitments are considered stronger than stated commitments found in strategies (Fox *et al*., 2011; 2015). We collected and analysed annual DAH (provided in 2021 constant US$) from all sources to HIV/AIDS, malaria and TB using the Institute for Health Metrics and Evaluation (2023) Financing Global Health visualization tool. DAH to address dengue, DD hepatitis, measles and meningitis is not tracked in the database. The diseases may be partially prioritized by DAH allocated to ‘child health’, including vaccines (e.g. rotavirus for DD), and ‘other infectious diseases-other’, but the categories are too broad to claim representation of priority for the untracked diseases. That DAH for these diseases is not specifically tracked likely indicates lower priority levels relative to HIV/AIDS, malaria and TB.

Bibliographic entries in the PubMed database of health and medical scholarship are a priority indicator featuring historical depth and relatively wide if not fully representative geographic breadth in the ‘scientific research arena’ (Bump *et al*., 2013; Smith *et al*., 2021). We searched PubMed (National Library of Medicine, 2023c) using the following MeSH terms to identify annual trends in research and publishing attention: dengue; diarrhoea OR infectious diarrhoeal disease; hepatitis; HIV infections; malaria; measles; meningitis; and tuberculosis. We also collected and analysed data on non-industry-sponsored clinical trials to measure priority in a sub-arena that focuses on conducting research on the efficacy of emerging biomedical, behavioural and other health interventions. Clinicaltrials.gov (National Library of Medicine, 2023a) is the largest international registry recording funding information. After observing that data were erratic during the registry’s first few years, we limited data collection to non-industry-funded trials started between 2004 and 2022. Search terms included: dengue; diarrhoea; hepatitis; HIV/AIDS; malaria; measles; meningitis; and tuberculosis. We also used clinicaltrials.gov to collect data on industry-funded trials—an indicator of priority in the ‘pharmaceutical industry arena’ (Fisher *et al*., 2015; Smith *et al*., 2021). In industry arenas, product development, supply and marketing may serve as other indicators of priority that are better suited to industry-specific and in-depth case study research.

We used publishing trends as an indicator of priority in the ‘news media arena’ (Hudacek *et al*., 2011; Smith *et al*., 2021). We searched the Access World News database for dengue, diarrhoea, hepatitis, HIV/AIDS, malaria, measles, meningitis and tuberculosis (Newsbank, 2023). The database covered news publications (total, *n* = 14 470) in seven regions at the time of data collection (November 2023): Africa (*n* = 678); Asia (*n* = 985); Australia/Oceania (*n* = 827); Europe (*n* = 1321); Middle East (*n* = 448); North America (*n* = 10 131); and South America (*n* = 80).

Turning to analysis of ‘patterns and scale of year-to-year change’, it is important to distinguish status quo change from the kind of large-scale change that represents departure from typical priority levels. Punctuated equilibria scholars define this kind of large-scale and relatively rare change as substantial deviation from the norm—the norm (status quo change) hovers around the mean (Baumgartner and Jones, 1993; Jordan, 2003; Breunig and Koski, 2006). We draw primarily on Jordan (2003), who set thresholds to determine when annual per cent change in budgets fell into a typical range of change category vs the large-scale and relatively rare punctuation category. More sophisticated statistical methods of analysis are available and might be used in the future, but require use of larger datasets than are currently available for application to global health priority (see Breunig and Koski, 2006; Martin and Streams, 2015, for instance).

Jordan (2003) set thresholds for punctuated change at multiples of mean annual change. She used annual budget ‘increases’ of >35% and ‘decreases’ of >25% to assess punctuation on the basis that incremental (often set at **≤**10% for budgets) increases are the norm; therefore, the threshold for punctuated decrease is lower. Using the same logic of typically increasing trends in resource allocations that are indicative of priority (e.g. more aid, publishing, clinical trials), we set conservative starting thresholds for ‘increases’ of ≥200% (double) and ‘decreases’ of ≥150% from mean annual change in priority for all resource allocations by arena. We set conservative thresholds (very large increases and decreases compared with mean change) to gain insights to how common large-scale change in priority is in the arenas. These thresholds may require adjustment as the evidence base grows. We applied these preliminary, conservative thresholds to indicators of priority within the international aid, scientific research, pharmaceutical industry and news media arenas, which feature large enough numbers for meaningful analysis of percent change.

This study advances the systematic measurement of patterns of priority for global health issues in relation to propositions informed by scholarship in a host of key agenda setting arenas. The work remains exploratory, however, and features several limitations. Resource allocations are considered strong priority indicators in global health (Ravishankar *et al*., 2009; Fox *et al*., 2011; 2015; Pitt *et al*., 2018; Smith *et al*., 2021). Priority in some arenas may be robustly measured using a single strong proxy (e.g. DAH) while multiple measures or a composite indicator may enhance accuracy for other arenas (e.g. industry). Analysis of priority in some arenas (e.g. news media and scientific research) is skewed towards greater high-income country than low- and middle-income country representation due to greater production and capture in databases. Interaction effects between arenas are reflected in findings. Lastly, the thresholds for analysis of large-scale change were set conservatively at multiples of mean (status quo) change—large-scale change, particularly the kind that qualifies as relatively rare punctuation, may thereby be underestimated in this preliminary exploration. Nonetheless, the study offers new insights to patterns of priority that are theoretically informed and contributes methods that may be used to study them at greater scale.

## Findings

A rational model based on mortality burden as opposed to a socially constructed model based on international norms offer competing logical explanations for the order of global health priorities. However, our findings are wholly consistent with neither (Tables 2 and 3). In none of the global health agenda setting arenas studied did priority align fully with the mortality burdens of eight infectious diseases or their prominence in major international development goals over time. The study findings presented in this section first illuminate the order of relative priority for eight communicable diseases in four agenda setting arenas between 2000 and 2022. The evidence detracts from Propositions 1 and 2 (rational model and international norms, respectively) and supports Proposition 3 (disease priorities are likely to vary by arena). Findings on differences in the scale of typical year-to-year change in resource allocations that are indicative of priority and the frequency of large-scale change are then presented. There is some support for Proposition 4 (overall resource allocations by arenas are likely to be characterized by patterns of primarily small-scale change that is occasionally punctuated by large-scale shifts) but not Proposition 5 concerning specific diseases. Propositions and support for them are summarized in Table 4.

**Table 2.** Disease order by mortality burden and arena priority during the MDG and SDG eras

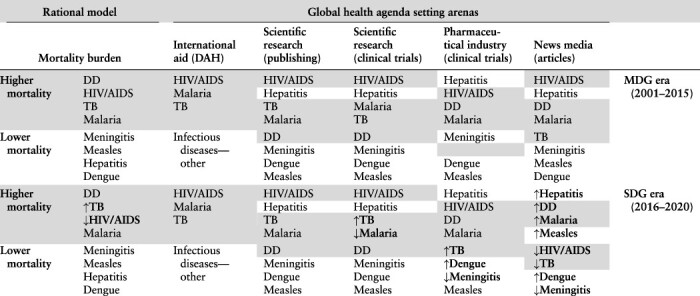

Key: The four diseases featuring the highest mortality burdens (>625 000 annual deaths) are shaded. Bold and arrows show change of order between time periods.

Notes: Ordered highest to lowest by indicator in two time periods (2001–2015 and 2016–2020, corresponding with the MDG and SDG eras). Clinical trials data are for 2006–2020 only. ‘Infectious diseases-other’ includes diseases for which DAH is not specifically tracked. Under the SDGs, the NTD Indicator covers dengue and waterborne diseases includes DD.

**Table 3.** Disease order by norm strength and priority in arenas during the MDG and SDG eras

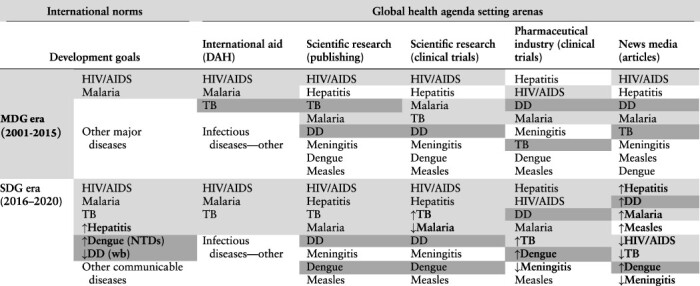

Key: Light grey = dedicated target or indicator; dark grey = well-recognized under a target or indicator; white = unspecified ‘other’. Bold and arrows show change of order between time periods.

Notes: Ordered highest to lowest by indicator in two time periods (2001–2015 and 2016–2020, corresponding with the MDG and SDG eras). Clinical trials data are for 2006–2020 only. ‘Infectious diseases-other’ includes diseases for which DAH is not specifically tracked. Under the SDGs, the NTD Indicator covers dengue and waterborne diseases (wb) includes DD.

**Table 4.** Evidence for propositions by arena and indicator

		Evidence by arena				
		International aid	Scientific research		Pharmaceutical industry	News media
Propositions		DAH	Publishing	Clinical trials	Clinical trials	Articles
**1**	Disease priority levels are likely to align with global disease burden.	Mixed	Weak	Weak	Weak	Weak
**2**	Disease priority levels are likely to align with major international development goals.	Mixed	Supported	Mixed	Mixed	Weak
**3**	Disease priorities are likely to vary by stakeholder arena.	Strong	Strong	Strong	Strong	Strong
**4**	‘Overall patterns of resource allocations’ from arenas are likely to be characterized by mostly small-scale change that is occasionally punctuated by large-scale shifts.	Small-scale	Punctuated equilibrium	Punctuated equilibrium	Volatile	Small-scale
**5**	‘Patterns of resource allocations from arenas to specific diseases’ are likely to be characterized by mostly small-scale change that is occasionally punctuated by large-scale shifts.	Volatile	Volatile	Volatile	Volatile	Volatile

### Relative priority for eight communicable diseases and examination of Propositions 1–3

**Figure 2.**
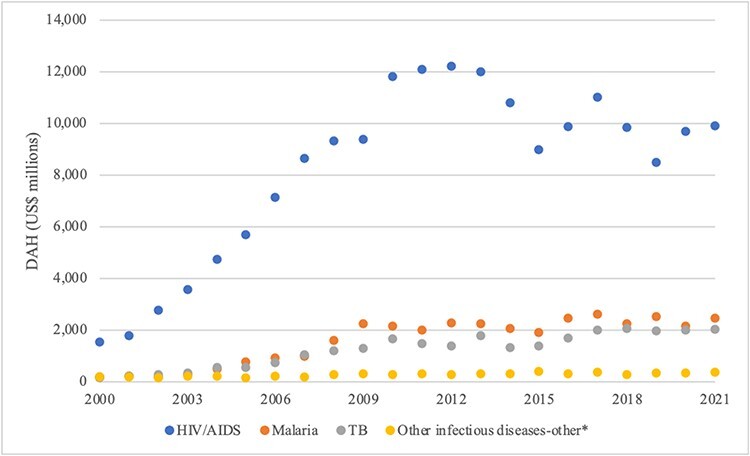
International aid arena: DAH, 2000–2021

Notes: DAH in constant 2021 US$. *‘Other infectious diseases-other’ includes all diseases for which DAH is untracked. The total for each year is divided by the number of untracked diseases included in this study to provide a very rough likely overestimate of allocations.

Source: Global Burden of Disease Collaborative Network (2020)].

In the ‘international aid arena’, allocations of DAH show HIV/AIDS was the most highly prioritized communicable disease since 2000 (Figure 2). DAH to malaria exceeded that to TB except for a brief period between 2003 and 2005. Funding streams for ‘other infectious diseases-other’, including highest burden DD, and lower burden meningitis, measles, hepatitis and dengue, were not specifically tracked in the Institute for Health Metrics and Evaluation (2023) database, suggesting they were not as highly prioritized in the arena.

The relatively high status of HIV/AIDS and malaria, which are among the highest burden infectious diseases and those most prominently featured among the MDGs and SDGs, provide some support for Propositions 1 and 2 (the rational model and international norms, respectively) (Tables 2 and 3). The lower status of the ‘other’ infectious diseases is also consistent with these propositions, with the exception of DD. The untracked status of DD, which poses the highest mortality burden among the eight diseases by far, is evidence against dominance of the rational model in explaining priority in the arena. The untracked status of hepatitis and to some degree NTDs (dengue) and waterborne diseases (DD) runs counter to international norms during the SDG era. In sum, the order of priorities in the international aid arena provide weak support for Propositions 1 and 2.

**Figure 3.**
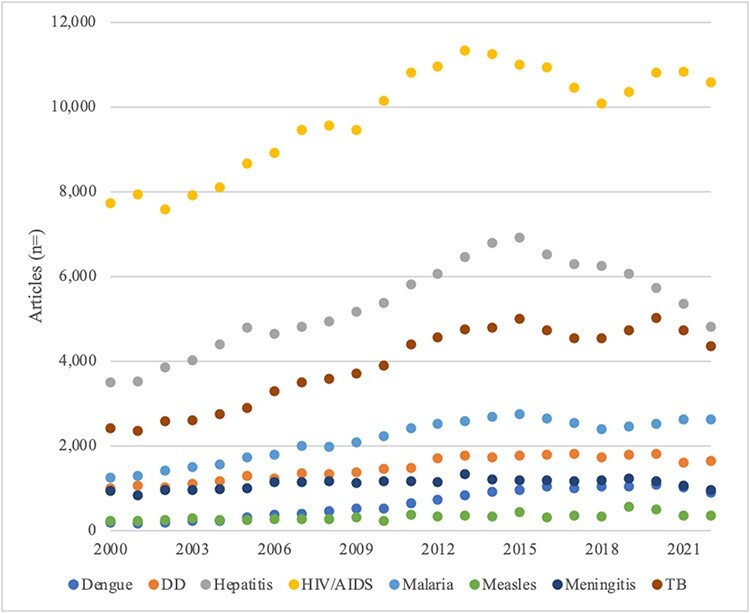
Scientific research arena: publishing, 2000–2022

Source: National Library of Medicine (2023c).

In the ‘scientific research arena’, bibliographic entries in PubMed reveal a highly consistent order of priority among the eight diseases between 2000 and 2022 (Figure 3). There is only one exception—at the bottom of the heap, research publishing priority for measles surpassed that for dengue by a slight margin between 2000 and 2004. Dengue gained somewhat greater traction thereafter but remained second to last place measles through 2022. Using ratios that cover the entire study period (2000–2022) to show relative research publishing priority, HIV/AIDS outpaced hepatitis by a ratio of 1.8 to 1, TB 2.5, malaria 4.5, DD 6.6, meningitis 8.9, dengue 15.4 and measles 31.1.

**Figure 4.**
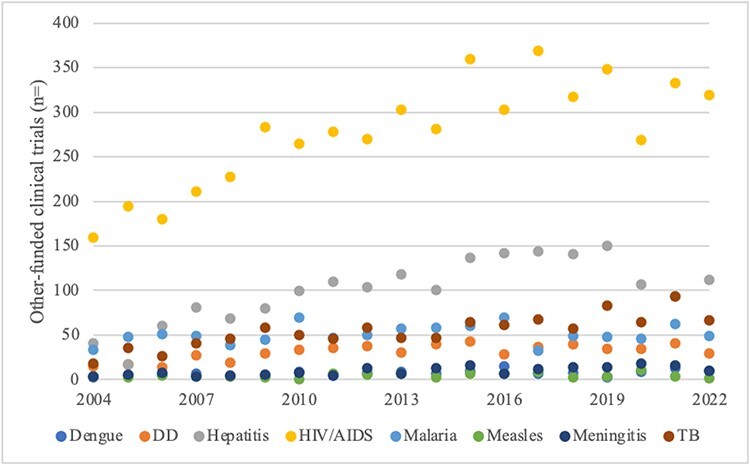
Scientific research arena: non-industry-sponsored clinical trials, 2004–2022

Source: National Library of Medicine (2023a).

Indicative of priority among actors conducting clinical trials in the scientific research arena, trials with public and non-profit funding sources largely show the same order of priority as research publishing (Table 2). Forming the only exception to the consistent order of priority, trials on malaria outnumbered those on TB (on average) through 2016 (Figure 4). In aggregate, non-industry-funded clinical trials on HIV/AIDS show substantial priority over the other diseases—ratios range from 2.8 to 1 over second place hepatitis to much higher multiples for those at the bottom (28.6 to 64.3 over meningitis, dengue and measles).

On publishing and clinical trials measures of priority in the scientific research arena, the order is inconsistent with the diseases’ relative mortality burdens (Table 2). Hepatitis ranked second on both priority measures along with HIV/AIDS, malaria and TB despite its relatively low mortality burden. By contrast, highest burden DD was consistently in the lower half. Overall, the evidence is against Proposition 1 (rational model). Turning to international norms, priority for HIV/AIDS and malaria was consistent with their relatively high status among the MDGs and SDGs. And, early attention to TB and hepatitis presaged their elevated place among the SDGs, gaining dedicated indicators (Table 3). The four diseases without dedicated targets and indicators rank in the lower half in both time periods. Overall, there is moderate support for Proposition 2.

**Figure 5.**
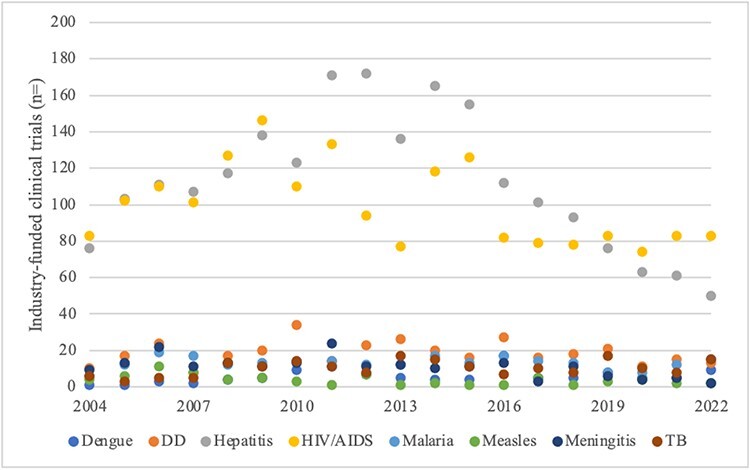
Pharmaceutical industry arena: industry-sponsored clinical trials, 2004–2022

Source: National Library of Medicine (2023a).

In the ‘pharmaceutical industry arena’, the order of priority for the four diseases with the most industry-funded clinical trials—hepatitis, followed by HIV/AIDS, DD and malaria—was highly consistent between time periods (Figure 5; Table 2). It varied over time among a tightly bunched lower half, though trials on measles persistently lagged all others. In aggregate, newly started industry-funded clinical trials on hepatitis slightly exceeded those on HIV/AIDS (1.1:1 ratio). Industry-funded clinical trials on hepatitis led the others by a wider margin: DD (6.1:1 ratio); malaria (8.6); meningitis (10.4); TB (11.0); dengue (19.4); and measles (30.0).

Though HIV/AIDS, DD and malaria are among the leaders for both burden and clinical trials, relatively low-burden hepatitis leads, departing from the rational model (Table 2). The priority order indicated by industry-funded clinical trials for the lower-burden diseases and higher-burdenTB also departs from the rational model, detracting from Proposition 1. Early priority for hepatitis broke from international development norms represented in the MDGs, but dovetailed with its status as SDG Indicator 3.3.4. Lower priority for meningitis, dengue and measles is consistent with their lower status among the major international development norms—as is higher status for HIV/AIDS and malaria. Higher priority for DD represents a departure from international norms. In sum, with DD relatively highly prioritized, evidence to support Proposition 2 in the pharmaceutical industry arena is mixed.

**Figure 6.**
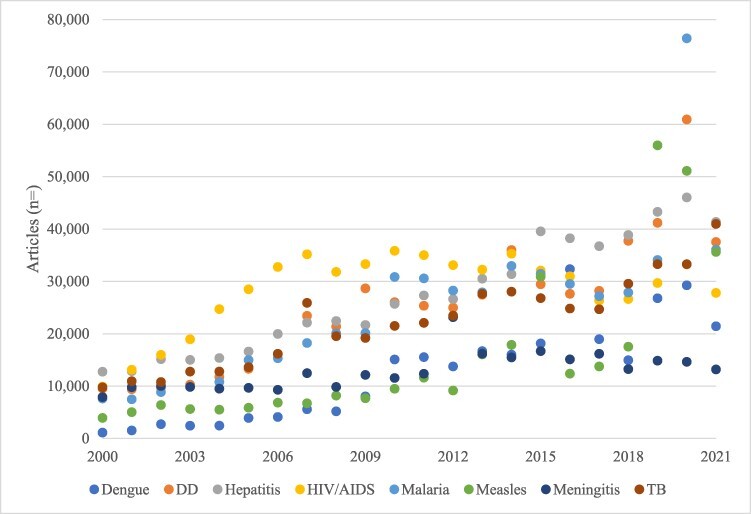
News media arena: articles, 2000–2021

Source: Access World News (Newsbank, 2023).

The volume of publishing on the eight diseases in the ‘news media arena’ shows a complete reshuffling of priorities over time (Figure 6). Among the leaders, HIV/AIDS received the most publishing attention each year from 2001 through 2013. DD replaced HIV/AIDS as the disease most highly prioritized for publishing in 2014 only to be displaced the next year by hepatitis. Hepatitis led until it was displaced by come-from-behind measles in 2019 and then malaria in 2020. More generally, between 2001 and 2015 (the MDG era), HIV/AIDS, hepatitis, DD, malaria and TB regularly received the most publishing attention, outpacing priority for the other diseases by wide margins. Between 2016 and 2020 (the SDG era), hepatitis, DD and malaria took the top three publishing priority spots (range, *n* = 194 967 to 203 042 articles) (Table 2). Measles, HIV/AIDS and TB clustered in a middle tier (range, *n* = 145 500 to 150 635 articles) while dengue and meningitis pulled up the rear (range, *n* = 73 932 to 122 183 articles).

Consistent with the rational model, highest burden DD was persistently in the top tier for news publishing attention—as were relatively high burden malaria, and between 2001 and 2015 HIV/AIDS and TB (Table 2). But relatively low-burden hepatitis’s recently high status while priority for HIV/AIDS and TB declined is inconsistent with the rational model. Hepatitis’s elevated status is more consistent with international development norms represented by SDG Indicator 3.3.4 (Table 3). However, priority for measles rose above HIV/AIDS and TB in the latter period—a departure from the normative framework represented by the SDGs and the status warranted by the disease’s mortality burden. Overall, contrary to Propositions 1 and 2, the order of priority in the news media arena varies from the diseases’ mortality burdens and international development goals status.

### Patterns of annual change in priority for eight communicable diseases and examination of Propositions 4–5

**Figure 7.**
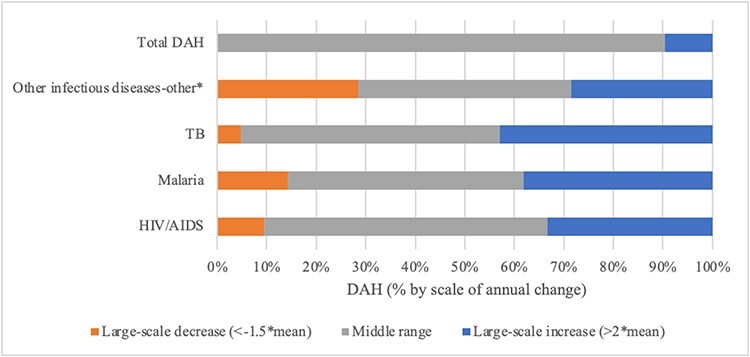
Scale of annual change in DAH by disease, 2000–2021

**Figure 8.**
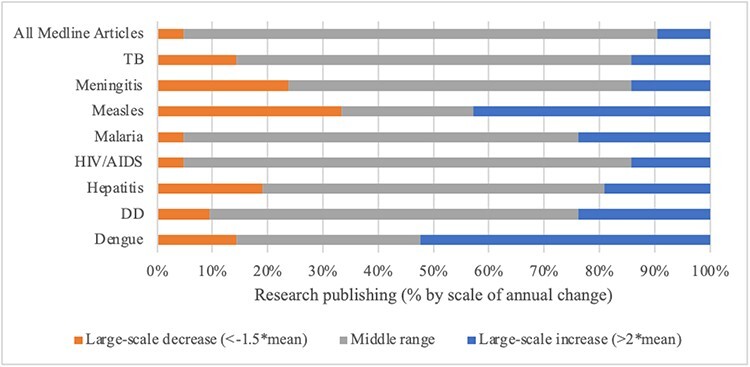
Scale of annual change in research publishing by disease, 2000–2022

**Figure 9.**
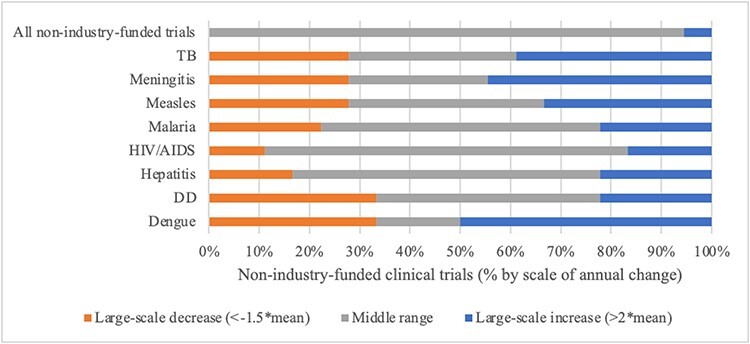
Scale of annual change in non-industry-funded clinical trials by disease, 2004–2022

**Figure 10.**
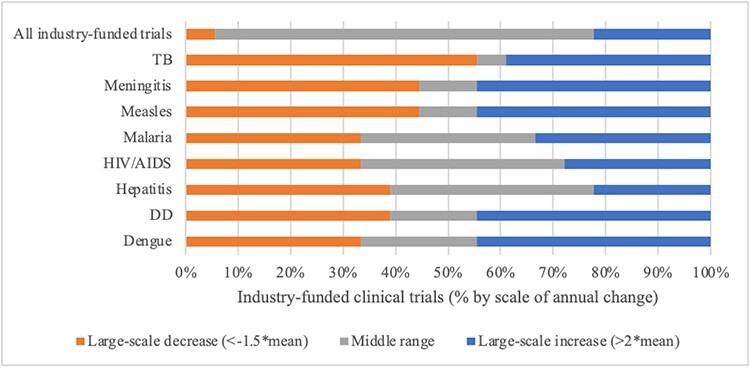
Scale of annual change in industry-funded clinical trials by disease, 2004–2022

**Figure 11.**
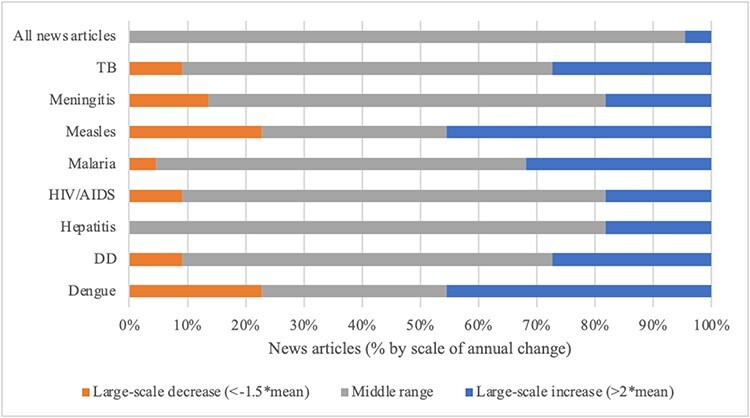
Scale of annual change in news media articles by disease, 2000–2022

Calculations of mean annual change in overall resource allocations and degrees of departure show differing patterns among the agenda setting arenas. Patterns of change at the disease level were consistently more volatile, with large-scale year-to-year change common. Figures 7–11 visually summarize findings for scale of annual change by arena and priority indicator for each disease alongside overall resource allocations in 100% stacked bar charts. Reading the figures left to right, in each bar: (1) orange sections show the proportion of ‘large-scale decreases’ in the given priority indicator; (2) grey sections show the proportion of middle range instances of ‘incremental change’; and (3) blue sections show the proportion of ‘large-scale year-to-year increases’. With rarity in large-scale changes the standard for patterns reflecting punctuated equilibrium, evaluative thresholds are set as follows: incremental patterns require more than 90% of change in the middle (mean) range; patterns consistent with punctuated equilibria require 80–90% of change in the middle range and 10–20% large-scale change; and patterns with less than 80% of change in the middle range are more volatile. Support for Propositions 4 and 5 is summarized in Table 4.

In the ‘international aid arena’, the dominant pattern of change in ‘overall’ DAH was small-scale—an incremental picture of stability (Figure 7)—with only two large-scale changes (increases of ∼24% in 2003 and ∼44% in 2020, the latter partly reflecting US$15.3 billion addressing the emergent COVID-19 pandemic). Annual change in DAH for HIV/AIDS, malaria, TB and those in the other infectious diseases-other category exhibited much more volatility. Changes in DAH allocations to HIV/AIDS, malaria and TB consisted primarily of large-scale increases and small-scale change, rarely losing significant ground. By contrast, DAH to other infectious diseases declined or increased sharply more frequently. Overall, patterns of DAH at the overall and disease levels ran counter to patterns of punctuated equilibria set forth in Propositions 4 and 5, respectively.

In the ‘scientific research arena’, annual changes in ‘overall’ publishing as captured by the Medline database were predominantly small in scale and punctuated by three instances of large-scale change (Figure 8), providing support for Proposition 4 (National Library of Medicine, 2023b). Running counter to Proposition 5, large-scale change in resource allocations indicative of priority dominated at the disease level. Research publishing on dengue and measles changed on a large-scale more frequently than it did for the other diseases—to somewhat greater advantage for dengue (with the balance increases) than for measles (which gained little ground) (Figure 3). Overall, patterns of priority as indicated by changes in publishing allocations at the disease level exhibited volatility, with substantial growth patterns for some (e.g. HIV/AIDS, hepatitis, TB).

Turning to non-industry-funded clinical trials as an indicator of priority in a subset of the ‘scientific research arena’, there was general stability in ‘overall’ resource allocations over time with one punctuation (supporting Proposition 4). Large-scale change was frequent at the disease level (Figure 9), detracting from Proposition 5. Patterns were particularly unstable for low-profile dengue and measles. Large-scale change was somewhat less frequent for high-profile HIV/AIDS and malaria, and for hepatitis, than for the others but still too frequent to qualify as rare punctuation.

Overall numbers of industry-funded registered clinical trials indicative of prioritization in the ‘pharmaceutical industry arena’ fluctuated widely, exhibiting a pattern of volatility (Figure 10). At the disease level, the vast majority of change was also large in scale. Large-scale change particularly dominated the landscape for TB, measles and meningitis, but patterns were universally volatile. Volatile patterns at the overall and disease levels contradict Propositions 4 and 5.

Lastly, the allocation of overall publishing resources in the ‘news media arena’ was highly stable on a year-to-year basis—there was just one large-scale increase (between 2005 and 2006). At the disease level, only hepatitis featured a pattern of change consistent with punctuated equilibrium (Figure 11). Volatility was the dominant pattern for all of the other diseases. The patterns detract from Propositions 4 and 5.

## Discussion

To summarize, patterns of year-to-year change in overall resource allocations varied by arena and were volatile at the disease level. Incremental change was more common at the overall level with patterns of stability or punctuated equilibria in the international aid, scientific research and news media arenas. Large-scale change was common overall and at the disease level in the pharmaceutical industry arena, revealing volatility in patterns of priority. Patterns of priority at the disease level were characterized by frequent large-scale change—volatility—for every indicator in every arena, evidence counter to Proposition 5.

This study offers new insights to the order and scale of changes in priority between 2000 and 2022 for a set of eight infectious diseases across four key global health agenda setting arenas in relation to five propositions from scholarship (Table 4). Only Proposition 3, suggesting priority is likely to vary by arena, is fully supported. Departing from prescriptions of competing rational models and socially constructed norms, it uncovers mainly misalignment with global mortality burdens and prominence in international development goals—likely due to interaction effects with other factors. In addition, this study introduces novel methods for analysing change in priority, advancing systematic measurement of status quo patterns vs the kind of large-scale change that may affect more substantial shifts in policy, programmes and health outcomes.

Evidence from this study mainly runs counter to Proposition 1, which suggests disease priority levels are likely to align with the global burden of disease. DD consistently posed a substantially higher mortality burden than any other disease included since 2000, but did not attract resource allocations proportionate to its burden in any arena. By contrast, relatively low-burden hepatitis was highly prioritized in three of the four arenas during the MDG and SDG eras. Relatively low-burden measles rose in the news media arena during the latter period without a substantial uptick in mortality burden through 2019. Measles prevalence, outbreaks and deaths are increasing, however (World Health Organization, 2023). It may be important to supplement operationalization of the rational model with other measures of threat to global health and even economic security as researchers seek to explain agenda setting dynamics that likely differ somewhat by arena.

The evidence in support of Proposition 2, suggesting disease priority levels are likely to align with major international development goals, is mixed. Priority for HIV/AIDS—headliner for MDG 6 and SDG 3.3—was high across the board except in the news media arena during the SDG era. Priority was also aligned with the international norms promoting malaria and TB in the scientific research and international aid arenas. Hepatitis was an early riser in the scientific research, pharmaceutical industry and news media arenas, presaging and perhaps facilitating its rise to prominence among the international development goals. Meningitis, measles and dengue—all without dedicated targets or indicators—tended to cluster near the bottom of the resource allocation priority list, but not universally. The mixed picture suggests international norms may have some explanatory power that is conditioned by other factors, and that agenda setting dynamics differ between arenas.

At the level of overall resource allocations by arena, findings on annual change provide limited support for Proposition 4. The study reveals that patterns of annual change in overall resource allocations were predominantly small in scale (incremental) in the international aid and news media arenas. Small-scale change was occasionally interrupted by large-scale change in priority in the research arena. In the pharmaceutical industry arena, large-scale change in the number of all clinical trials on a year-to-year basis was common. This could reflect general volatility in investments in clinical trials, patterns of successful and failed trials or other limitations of the indicator (Fisher *et al*., 2015). Composit indicators tailored to industry (e.g. medicines, unhealthy commodities) may offer more robust measurement of priority and patterns of change. Examination of such alternative measures is beyond the scope of this inquiry, but is part of the broader Global Health Agendas Project research programme (Parashar *et al*., working paper). These findings depart from previous scholarship, which advances punctuated patterns of change in public policy and global health (Baumgartner, Jones and Mortensen, 2018; Baumgartner and Jones, 1993; Shiffman *et al*., 2002; Hoffman, 2010; Martin and Streams, 2015).

At the disease level, our findings run completely counter to the patterns of punctuated equilibria encapsulated in Proposition 5. Large-scale change in priority for the infectious diseases as defined in this study (multiples of mean change) was very common in all arenas. Punctuation is defined by rare, large-scale change amidst a sea of stability. We observed patterns that were highly stable or consistent with punctuated equilibrium at the level of overall resource allocations in three arenas while volatility dominated at the disease level. Observation of the pattern across global health agenda setting arenas is unprecedented. It begs further inquiry and explanation. Overall, the contradictory findings raise the need for analyses covering a larger number of global health problems and offering insights to causal dynamics behind patterns of small- and large-scale change by stakeholder arena—because though there are likely some common factors (such as disease burden and international norms), other factors appear to interact and shape differences among global health agenda setting arenas.

## Conclusion

This study further develops a recently proposed arenas model for global health agenda setting, advancing more robust inquiry into how and why priority levels may vary over time. It challenges existing notions about patterns and scale of policy change over time and prominent models for explaining them. It shows how propositions concerning priority can be investigated through theoretically informed issue selection—using rational, normative and other models. It offers new, systematic methods for investigating patterns over short and long time horizons. These developments are crucial for scholars and proponents working to understand the complex web of interactions—those mechanisms of change—that determine why some problems attract greater resource allocations from a fuller range of relevant stakeholders than others in global and national governance contexts.

Empirical research covering a fuller range of global health issues is needed to determine whether the patterns revealed by this study are typical or exceptional, and what else shapes them. Directions for future research include:

- Examining the power of simultaneous alignment on mortality burden and prominence among major international development goals to determine priority levels in global health agenda setting arenas.
- How consistently are priorities in the international aid arena aligned with major international development goals? How much do such norms matter in other arenas? Do they have exclusionary (crowding out) effects?
- How do patterns of priority for global health problems track with relative mortality burdens compared with other measures consistent with rationality, such as morbidity and economic and security threats?
- In the pharmaceutical industry arena, how do potentially conflicting profit and social responsibility motives shape priorities? Towards more effective measurement, how robust are clinical trials in representing pharmaceutical industry priorities?
- Given that patterns of priority break with the global disease burden and to some extent international norms in the scientific research arena, what roles do budgetary, ethical and other factors play?
- Recognizing that the scale of change in resource allocations might differ between arenas, what is typical vs the kind of large-scale change (punctuation) that could alter an issue’s trajectory?

Further research is also needed to develop more robust ways of systematically analysing priority for health in other arenas and across other factors.

Larger-n studies should be complemented by medium-n and in-depth case study research that is better suited for analysing complex causal dynamics and evaluating the robustness of priority indicators by arena (Baumgartner and Jones, 1993; Dowding *et al*., 2016). This study advances inquiry into theoretically informed and systematic measurement of global health priorities within a subset of key stakeholder arenas. A strong foundational body of scholarship on global health agenda setting is positioned to inform deeper causal analysis (see Smith and Shiffman, 2018). Explanations for priority are likely to differ by arena and type of issue (e.g. infectious vs non-communicable diseases). This study helps to position scholars and other analysts to conduct more robust inquiry into causal dynamics in global and national health agenda setting contexts.

## Data Availability

Most of the data underlying this study are freely publicly accessible. Development assistance for health data are available from Institute for Health Metrics and Evaluation under Financing Global Health at https://vizhub.healthdata.org/fgh/. Data informing the analysis of health publishing are available from the National Library of Medicine at https://www.ncbi.nlm.nih.gov/pmc/. Clinical trials data are available from National Library of Medicine at https://www.ncbi.nlm.nih.gov/pmc/. Data informing the media priority analysis come from the Access World News database and were obtained under license from Newsbank (https://www.newsbank.com/) through Virginia Tech Libraries.

## Reflexivity statement

This project was carried out by a diverse, multiregional research team that includes experienced and emerging scholars.
